# Prenatal Diagnosis and Postnatal Findings of Cephalothoracopagus Janiceps Disymmetros: A Case Report

**DOI:** 10.1155/2012/273526

**Published:** 2012-05-29

**Authors:** Lívia Teresa Moreira Rios, Edward Araujo Júnior, Luciano Marcondes Machado Nardozza, Daniela Cristina Nacaratto, Antonio Fernandes Moron, Marília da Glória Martins

**Affiliations:** ^1^Mother-Child Unit, Universitary Hospital, Federal University of Maranhão (UFMA), 65020-560 São Luiz, MA, Brazil; ^2^Department of Obstetrics, Federal University of São Paulo (UNIFESP), 05303-000 São Paulo, SP, Brazil

## Abstract

Conjoined twins are rare variants of monozygotic twins, which result from an incomplete division of the embryonic disk. Cephalothoracopagus is a rare twin pregnancy described as imperfect fusion of the head and chest, but separated columns, limbs, and pelvis. They occur with incidence rates that range from 1 per 50,000 to 1 per 100,000 births; however, the incidence of the cephalothoracopagus variety is 1 per 58 conjoined twins. In the case of identical and symmetric faces caused by the orientations of the 2 notochordal axes that are perfectly ventroventral, they are called janiceps disymmetros. We present a prenatal diagnosis of a typical case of cephalothoracopagus janiceps disymmetros and the diagnostic confirmation by image and pathology exams.

## 1. Introduction

Twin is a phenomenon resulting from the fertilization of two ovules by two sperms (dizygotic) or the targeting of an embryo resulting from fertilization of one ovule by one sperm (monozygotic) [1, 2]. The dizygotic twin is constrained by natural conditions (heredity, race, maternal age, and parity) and artificial (assisted reproduction) [1–3]. The monozygotic twin, in turn, does not depend on any condition occurring independently and randomly in the approximate ratio of 1 : 25 births [1–3].

When the segmentation of the embryo occurs after the 13th day of fertilization, there is no complete separation between individuals, and the pregnancy occurs with the formation of a pair of conjoined twins, which is called conjoined twin, which by design will occur in the same placenta and a single amniotic cavity [1–3].

The embryological classification of conjoined twin described by Spencer [4] is as follows: (1) ventral union (87%)—cephalopagus (11%, top of head to umbilicus); thoracopagus (19%, conjoined heart); omphalopagus (18%, including lower thorax); ischiopagus (lower abdomen and genitourinary system); parapagus (pelvis and variable trunk); (2) dorsal union (13%)—craniopagus (5%, cranial vault); rachipagus (2%, vertebral column); pygopagus (6%, sacrum).

Cephalothoracopagus is a rare twin pregnancy described as imperfect fusion of the head and chest, but separated columns, limbs, and pelvis. It occurs once in every 58 sets of conjoined twins or once in every three million births [5]. The term janiceps is derived from Janus, the two-faced Roman god. This term is used in cases that the facial structures, anterior and posterior ones, are composed by each individual, who looks in the opposite directions. When the two faces are identical and symmetrical, this is named cephalothoracopagus janiceps disymmetros [6]. We present a prenatal diagnosis of a typical case of cephalothoracopagus janiceps disymmetros and the diagnostic confirmation by image and pathology exams.

## 2. Case Presentation

**Figure 1:**
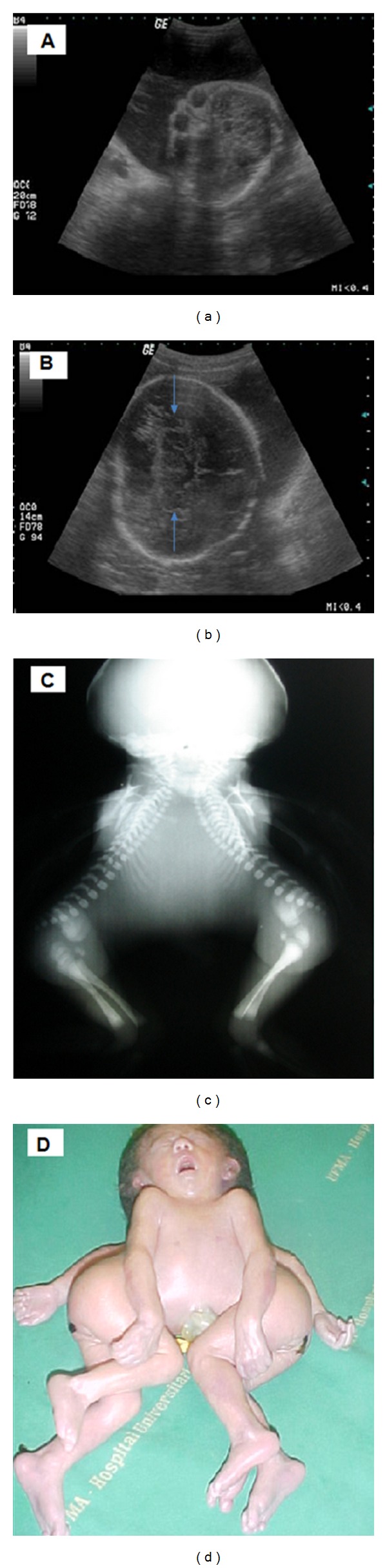
Cephalothoracopagus janiceps disymmetros. (a) Prenatal two-dimensional ultrasound showing only a skull with two faces and two eyes. (b) Prenatal two-dimensional ultrasound showing the brain with the thalamus fusion (blue arrows). (c) Postnatal X-ray showing the fusion of twins from head until upper abdomen. (d) Postnatal *postmortem* analysis confirming the prenatal diagnosis.

A 17-year-old primigravida, 31 weeks of pregnancy, was refereed to our hospital presenting a fetus with a large head circumference, one chest, and two vertebral columns. Two-dimensional (2D) ultrasound scan realized with a Voluson 730 Pro machine (General Electric, Medical System, Healthcare, Zipf, Austria) equipped with a volumetric convex probe (RAB 4–8 L) showed a conjoined twin-cephalothoracopagus janiceps disymmetros, one placenta, and polyhydramnios. Twins were fused from head until upper abdomen at the level of the umbilical cord; they had a single chest, a common liver, two vertebral columns, and two hearts. There was one skull with two faces (Figure 1(a)), one of them well-formed with two eyeballs, brain, and duplicates and fused thalamus (Figure 1(b)). After preterm labor at 34 weeks, twins weighed 1, 660 g and were born by Cesarean section, surviving for twenty minutes. The X-ray and* postmortem* analysis confirmed the prenatal diagnosis (Figures 1(c) and 1(d)).

## 3. Discussion

The prenatal diagnosis of cephalothoracopagus janiceps disymmetros is very important to the counseling of parents of poor neonatal prognosis. The diagnosis usually is realized by 2D ultrasound, as our case [7–9] in the end of second trimester. The 2D ultrasound is the main imaging method for diagnosis of fetal malformations, because it is cheap, without risk to women pregnant, and it is available in several secondary services. The magnetic resonance imaging (MRI) is an important method, but it is expensive, and it has some contraindications as metallic implants and claustrophobia. The three-dimensional (3D) ultrasound permits a spatial visualization of fetal malformation; beyond better understanding of fetal malformation by parents, but until now, the 3D ultrasound did not prove to be better than 2D ultrasound in the diagnosis of fetal malformations.

Our case was diagnosed in the third trimester because the woman pregnant started late the prenatal care, and she realized only an ultrasound scan at 29 weeks that evidenced the fetal malformation. There are in the literature cases of early diagnosis of cephalothoracopagus janiceps disymmetros [10], or using other imaging methods as MRI [7, 11] and 3D ultrasound [4, 6]. The advantage of 3D ultrasound in the rendering mode is to permit better understanding of the anomaly by parents, facilitating counseling [6]. The MRI was not realized because the woman pregnant arrived late in our service. The 3D ultrasound was not realized because it would not add new information already provided by 2D ultrasound and because the interruption of pregnancy in cases of fetal malformations is not permitted by law in Brazil.

 However, both methods are complementary to 2D ultrasound, being this considered the gold standard to the prenatal diagnosis of cephalothoracopagus janiceps disymmetros. In our case, the diagnosis was proven by X-ray and macroscopic *postmortem *analysis, similar to what was described by Slager et al. [5]. The X-ray showed the fusion of twins from head until upper abdomen. The *postmortem* analysis showed only a skull with two eyes, two ears, and one nose. The necropsy was not realized because the parents did not permit it.

Although the prognosis is extremely poor and surgical separation of the complex craniofacial defects is usually not offered [8], the early diagnosis is important to counseling of parents about the interruption of pregnancy, in countries where the interruption of pregnancy is permitted. The mode of delivery is usually by Cesarean section because dystocia is a potential complication. Before 24 weeks, termination of pregnancy by the vaginal route with destructive procedures is an option. After 24 weeks' gestation, termination by hysterotomy was seen more prudent [12].

Unfortunately, this patient was referred to our service at 31 weeks of pregnancy. Maranhão is a poor state of the Northeast of Brazil, and usually the public prenatal care to start in the second trimester of pregnancy and the first ultrasound scan is realized at the end of second trimester. It is common that several fetal malformations arrive in the reference services in the third trimester, as this type of conjoined twin. Unfortunately, Brazil is the greatest catholic country of the world, and the interruption of pregnancy is permitted only in cases of sexual violence or high risk of death to the pregnant woman. Interruption of pregnancy in cases of fetal malformations (the art. 128 of decree-law no. 2848 of December 7, 1940), even in cases with incompatible life in the postpartum period, is not permitted by law.

In summary, cephalothoracopagus janiceps disymmetros is an uncommon type of conjoined twin, and the prenatal diagnosis is very important to counseling of parents because of poor neonatal prognosis and the possibility of interruption of pregnancy. However, in Brazil and other countries, the interruption of pregnancy by fetal malformation is not permitted by law. This law is legal but maybe it is immoral, because it submits the pregnant woman to a pregnancy without fetal prognosis and to the risks of continuing pregnancy as hypertensive disorders, polyhydramnios, and abruption placentae. These complications can increase the maternal morbidity and mortality.
